# Facial Tuberculoid Leprosy

**DOI:** 10.4269/ajtmh.21-0696

**Published:** 2022-03-21

**Authors:** Niraj Parajuli, Sujan Shrestha

**Affiliations:** ^1^ Department of Dermatology & Venereology, National Academy of Medical Sciences, Bir Hospital, Kathmandu, Nepal;; ^2^ Department of Pathology, National Academy of Medical Sciences, Bir Hospital, Kathmandu, Nepal

**Figure 1.**
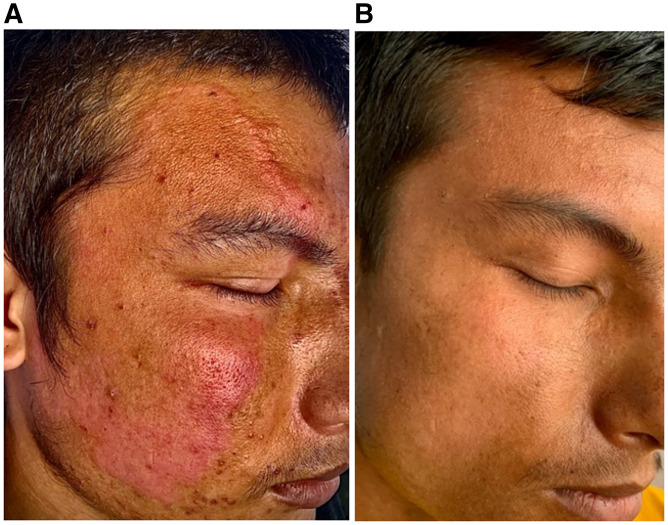
(**A**) A large erythematous plaque over right face. (**B**) Complete resolution after 6 months of multidrug therapy. (Patient consent was taken for the photos.) This figure appears in color at www.ajtmh.org.

**Figure 2.**
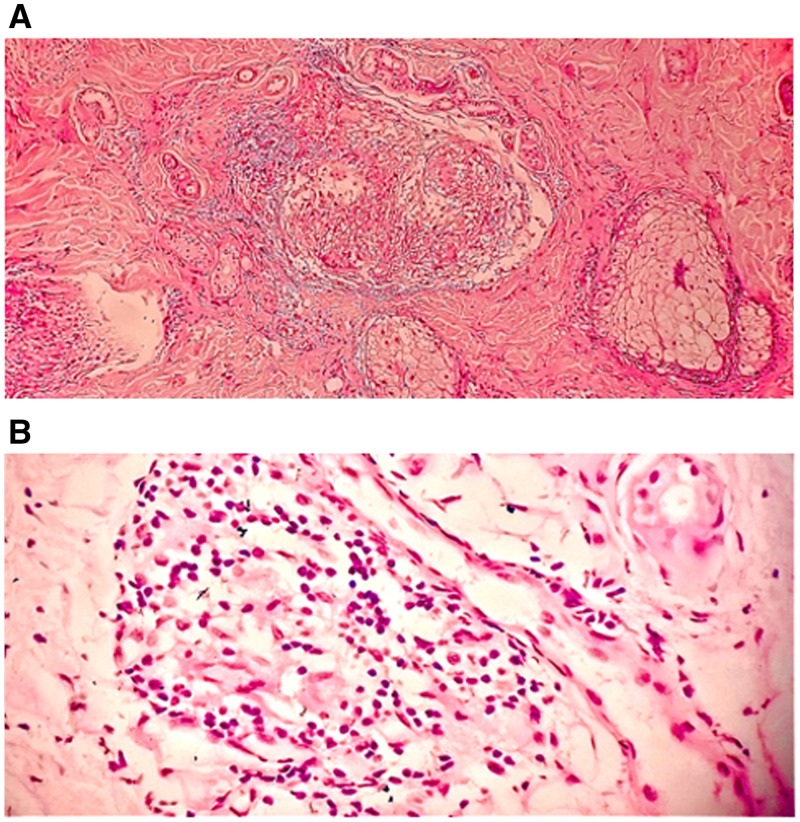
(**A**) A low-power magnification showing multiple well-formed granulomas in the dermis (hematoxylin and eosin stain 20× magnification). (**B**) A high-power magnification showing granuloma and perineural inflammation (hematoxylin and eosin stain 40× magnification). This figure appears in color at www.ajtmh.org.

A 25-year-old male presented with an asymptomatic erythematous enlarging plaque over the right face for a duration of almost 3 years. Patient was misdiagnosed and treated as a case of tinea faciale multiple times during this 3-year period. On examination, there was a single erythematous plaque over the right side of the face (Figure 1A). Both cold and pain sensations were decreased over the lesional skin. Nontender enlargement of right supraorbital nerve was also noted. Slit-skin smear from lesion stained with modified Ziehl-Neelson stain did not reveal any acid-fast bacilli. A punch biopsy from the border of the plaque and stained with hematoxylin and eosin stain showed aggregates of epitheloid cells rimmed by lymphocytes and multinucleated giant cells in the dermis, with perivascular and perineural lymphocytic infiltration suggesting tuberculoid leprosy (Figure 2A and B). Patient was started on multidrug therapy (MDT) with rifampicin, dapsone, and clofazimine. The plaque completely disappeared after 6 months of treatment with MDT (Figure 1B).

Leprosy is a chronic disease caused by *Mycobacterium leprae*. It has a long incubation period ranging from months to years. It can present with various morphologies, which depends on the bacilli load and host immunity.^1^^,^^2^ Loss of sensation over the lesional skin is one of the cardinal sign of leprosy, but the sensation in the facial leprosy lesion may be intact due to the abundant sensory nerves. Biopsy is useful in making a diagnosis in difficult cases when slit skin smear is negative. The histopathological features of tuberculoid leprosy are the presence of well-formed granulomas with lack of acid-fast bacilli.^3^ The prominent erythema and irregularity of the lesion might be due to the topical steroid use. Leprosy was eliminated as a public health problem from Nepal in 2010. However, new cases are being detected at a constant rate. This case highlights a rare presentation of only a single facial leprosy plaque which was the main reason for late diagnosis.
